# Tumor Microenvironment-Responsive Polymeric Nanocarriers for the Treatment of Triple-Negative Breast Cancer

**DOI:** 10.3390/pharmaceutics18091083

**Published:** 2026-08-28

**Authors:** Adnan Murad Bhayo, Ying Li, Alaa R. Aboushanab, Junyi Lin, Ashkan Hassankhanirad, Wei Li, Jingjing Sun

**Affiliations:** 1 Department of Chemistry, University of Prince Edward Island, 550 University Ave, Charlottetown, PE C1A 4P3, Canada; ambhayo@upei.ca; 2 Department of Pharmaceutical Sciences, College of Pharmacy, University of Nebraska Medical Center, Omaha, NE 68106, USA; yingli@unmc.edu (Y.L.); aaboushanab@unmc.edu (A.R.A.); julin@unmc.edu (J.L.); ahassankhanirad@unmc.edu (A.H.); chli@unmc.edu (W.L.); 3 Fred & Pamela Buffett Cancer Center, University of Nebraska Medical Center, Omaha, NE 68106, USA

**Keywords:** stimuli-responsive polymers, triple-negative breast cancer, tumor microenvironment, polymeric nanocarriers, drug delivery

## Abstract

Triple-negative breast cancer (TNBC) is an aggressive and heterogeneous subtype lacking effective targeted therapies. Its tumor microenvironment (TME) exhibits distinct features, including acidity, redox imbalance, elevated reactive oxygen species (ROS), and hypoxia, which provide exploitable triggers for targeted drug delivery. Stimuli-responsive polymeric nanocarriers have emerged as promising platforms that enable spatiotemporally controlled and site-specific therapeutic release in response to these endogenous cues, as well as exogenous stimuli such as temperature and light. These systems improve drug accumulation, penetration, and therapeutic efficacy while reducing systemic toxicity. Unlike previous reviews that broadly discuss nanocarriers in cancer therapy, this review focuses on the structure–function relationships of TME-responsive polymeric systems in TNBC and their translational limitations. We summarize recent advances in pH-, redox-, ROS-, hypoxia-, photo- and temperature-responsive polymers, highlighting their design strategies and therapeutic applications. Key challenges, including stimulus heterogeneity, limited in vivo validation, and clinical translation barriers, are also discussed. This review provides a concise framework for the rational design of programmable, multi-responsive polymeric nanomedicines for TNBC therapy.

## 1. Introduction

Triple-negative breast cancer (TNBC) is a biologically aggressive and clinically challenging subtype of breast carcinoma, defined by the absence of estrogen receptor (ER), progesterone receptor (PR), and human epidermal growth factor receptor 2 (HER2) expression [1,2,3]. Accounting for approximately 15–20% of breast cancer cases, TNBC is associated with high histological grade, genomic instability, early metastatic dissemination, and poor clinical prognosis [4,5]. In contrast to hormone receptor-positive and HER2-amplified tumors, TNBC lacks well-defined molecular targets, restricting systemic treatment largely to cytotoxic chemotherapy, with emerging roles for immune checkpoint inhibitors and poly (ADP-ribose) polymerase (PARP) inhibitors [6]. Despite these advances, therapeutic resistance, rapid disease progression, and dose-limiting toxicity remain major clinical challenges, highlighting the urgent need for more precise and tumor-selective treatment strategies [7].

Increasing evidence indicates that the clinical behavior of TNBC is not solely dictated by intrinsic tumor cell properties but is profoundly influenced by the tumor microenvironment (TME) [8]. The TME is a complex and dynamic ecosystem composed of cancer-associated fibroblasts, immune cells, extracellular matrix (ECM), and aberrant vasculature, which collectively regulate tumor progression, metastasis, immune evasion, and response to therapy [9,10]. TNBC tumors are frequently characterized by high immune cell infiltration accompanied by functional immune suppression, extensive stromal remodeling, and abnormal vascular architecture, all of which contribute to heterogeneous drug distribution and limited therapeutic efficacy [11,12,13,14,15]. These features establish the TME not merely as a passive structural component but as a critical determinant of disease progression and therapeutic response.

Importantly, the TNBC microenvironment exhibits a range of distinct biochemical and biophysical characteristics that can be exploited for targeted therapeutic intervention. These include extracellular acidosis resulting from metabolic reprogramming [16,17], elevated intracellular glutathione levels and redox imbalance [18,19], increased oxidative stress with accumulation of reactive oxygen species (ROS) [20], and regions of hypoxia driven by abnormal vascularization [21,22,23]. In addition, enzymatic dysregulation, particularly the overexpression of matrix metalloproteinases, contributes to extracellular matrix remodeling and tumor invasion, providing enzyme-specific triggers for localized drug release [24,25,26]. External stimuli, such as temperature and light, can further be leveraged to achieve spatiotemporal control of therapeutic activation [27,28]. Collectively, these TME-associated features offer multiple orthogonal triggers for the development of responsive drug delivery systems capable of selective activation within tumor tissues.

Polymeric nanomaterials provide a highly versatile platform for translating these biological cues into precision therapeutic strategies [29,30,31]. Their structural tunability and functional adaptability enable the rational design of nanocarriers with controlled physicochemical properties, including size, surface charge, stability, and responsiveness. By incorporating stimuli-sensitive linkages, charge-switching mechanisms, and multi-functional domains, polymeric systems can be engineered to undergo sequential processes such as prolonged circulation, tumor accumulation, cellular internalization, and intracellular drug release [32,33,34,35,36,37,38,39,40]. Such programmable behavior is particularly advantageous in overcoming the multiple biological barriers associated with TNBC, including poor tumor penetration, intratumoral heterogeneity, and resistance to conventional therapies. However, the complexity of TME heterogeneity, variability in stimulus intensity, and concerns related to polymer safety, degradation, and large-scale production continue to limit clinical translation [41].

Despite promising preclinical results, the clinical translation of polymeric stimuli-responsive nanocarriers still faces several challenges. Long-term safety requires careful evaluation of biodistribution, clearance, immunogenicity, biodegradation, and potential toxicity following repeated administration [42]. Reproducibility during synthesis and formulation is another major challenge, as variations in particle size, drug-loading efficiency, and drug-release behavior may affect product quality and therapeutic performance [43]. In addition, sterilization strategies compatible with Good Manufacturing Practice (GMP) must preserve nanoparticle stability and biological activity [44]. Finally, comprehensive physicochemical characterization, quality control, stability testing, and toxicological evaluation are required to meet regulatory standards before clinical translation [45]. Beyond these translational considerations, current research also has several scientific limitations. Studies remain fragmented and often focus on individual triggers without fully integrating TME biology into polymer design, particularly in the context of TNBC. Moreover, key challenges, including stimulus heterogeneity, the limited predictive value of preclinical models, and the scalability of polymer synthesis, remain insufficiently addressed.

To provide a focused and balanced overview of this rapidly evolving field, this review primarily considers studies published between 2020 and 2026 that are directly relevant to TME-responsive polymeric nanocarriers for TNBC therapy. Earlier landmark studies were also included where necessary to introduce the fundamental principles of stimuli-responsive polymers and the biological characteristics of the TNBC tumor microenvironment. The literature was selected based on its relevance to the design, synthesis, TME responsiveness, characterization, cargo delivery, therapeutic efficacy, structure–property relationships, and translational potential of polymeric nanocarriers in TNBC models. Studies not directly related to polymeric nanocarriers, TME-responsive systems, or TNBC, or those lacking sufficient information on system design or therapeutic application, were excluded. Particular emphasis was placed on studies that provide meaningful insights into polymer design, TME responsiveness, therapeutic efficacy, and current limitations.

The review is organized according to the major TME-responsive strategies, including pH-, redox-, ROS-, hypoxia-, photo-, and temperature-responsive systems. Within each category, studies are critically discussed in terms of their design rationale, structure–property relationships, therapeutic applications, and limitations to facilitate comparisons across different polymeric platforms. In addition, a dedicated section on polymer design considerations for clinical translation has been included to discuss key factors affecting the development of clinically relevant polymeric nanocarriers, including biocompatibility, biodegradability, degradation behavior, degradation-product toxicity, molecular weight, physicochemical properties, and structural stability. This section also highlights broader issues related to safety, reproducibility, and translational feasibility beyond stimulus-responsive therapeutic performance.

Accordingly, this review provides a comprehensive and critical overview of recent advances in TME-responsive polymeric nanocarriers for TNBC, highlighting current challenges and key considerations for their future clinical translation.

## 2. pH-Responsive Polymers

The acidic TME of TNBC, with extracellular pH values of approximately 6.2–6.9, provides a distinct biochemical trigger for selective therapeutic delivery [46,47]. pH-responsive polymers exploit this acidity to achieve controlled drug release and enhanced cellular internalization while remaining stable under physiological conditions (pH 7.4) [48,49]. These systems are typically designed with ionizable moieties and acid-labile linkages that undergo protonation, bond cleavage, or structural destabilization in acidic environments. Protonation within endosomal compartments can further promote endosomal escape through the proton sponge effect, while surface charge modulation under acidic conditions enhances interactions with cell membranes, thereby improving cellular uptake [50,51,52,53,54]. However, it should be noted that extracellular acidity in tumors is often heterogeneous, which may limit the predictability of pH-triggered activation in vivo.

**Figure 1:**
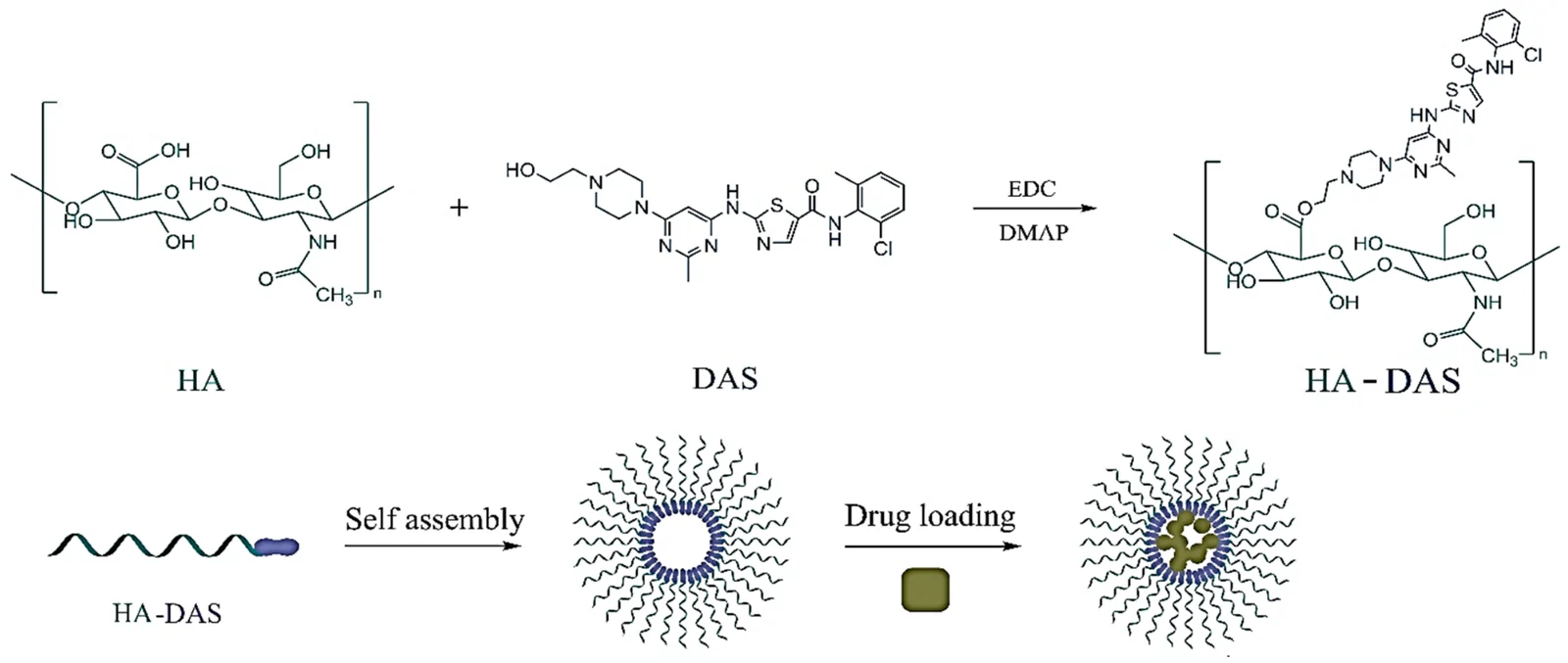
Synthesis route of the pH-responsive prodrug copolymer (HA-DAS), followed by self-assembly and drug loading process [55]. Reproduced with Permission, Creative Commons CC-BY-NC, Taylor & Francis-2022.

Zeng et al. [55] developed pH-responsive hyaluronic acid-dasatinib polymeric nanoparticles to enhance the antitumor efficacy of dasatinib (Src family kinase inhibitor) and olaparib (PARP inhibitor) in TNBC. Dasatinib was covalently conjugated to hyaluronic acid through acid-labile ester bonds using EDC/DMAP chemistry, forming an amphiphilic polymer prodrug that self-assembles into nanoparticles with a hydrophilic hyaluronic acid shell and a hydrophobic dasatinib core. Olaparib was physically loaded into the hydrophobic core (Figure 1). In vitro release studies demonstrated stability at pH 7.4 but accelerated drug release under acidic conditions, confirming efficient pH-triggered ester bond cleavage. The nanoparticles exhibited enhanced cellular uptake, increased intracellular drug accumulation, and improved cytotoxicity in MDA-MB-231 cells. In vivo studies further showed significant tumor inhibition with reduced systemic toxicity, indicating effective tumor-selective drug delivery. This design is particularly relevant for TNBC due to CD44 overexpression, which enables hyaluronic acid-mediated targeting.

Lu et al. [56] reported pH-responsive polymeric micelleplexes based on PEG-*b*-PDMAEMA-b-PDPA triblock copolymers for co-delivery of doxorubicin (DOX) and Bcl-2 siRNA. The system demonstrated high drug loading and siRNA condensation efficiency and exhibited rapid destabilization as pH decreased from 7.4 to 5.0, consistent with protonation of PDPA and PDMAEMA segments. In vitro studies showed that Bcl-2 knockdown sensitized drug-resistant TNBC cells to DOX, resulting in enhanced cytotoxicity compared with control treatments. These results highlight the advantage of pH-responsive systems in enabling synergistic chemo-gene therapy through coordinated intracellular release.

Jin et al. [57] developed PEG-detachable pH-responsive nanoparticles based on a PEI-PLA/PEG-PAsp system for co-delivery of paclitaxel (PTX) and VEGF siRNA. The system utilizes the proton sponge effect of PEI to induce endosomal rupture under acidic conditions, facilitating cytoplasmic delivery of siRNA while avoiding lysosomal degradation. This dual-delivery approach enables simultaneous cytotoxic and anti-angiogenic effects, resulting in enhanced tumor growth inhibition and apoptosis compared with single-agent treatments.

Hu et al. [58] reported pH-responsive PEG-modified PLGA nanoparticles coated with a tannic acid-iron(III) complex (DOX-TPLGA NPs) for controlled DOX delivery. The Fe^3+^-TA shell undergoes disassembly under acidic conditions, leading to enhanced drug release compared with non-responsive systems. These nanoparticles showed improved cellular uptake, reduced IC_50_ values, and significant tumor inhibition in TNBC models, with minimal systemic toxicity.

Scialla et al. [59] developed pH-responsive polymer-coated magnetic lipid nano-vehicles (AS_mLNVs-DOX) for targeted therapy of TNBC through targeting tumor-associated macrophages (TAMs). The nanovehicles comprised a Carnaúba wax lipid core encapsulating iron oxide nanoparticles and DOX, surface-modified with mannose ligands and acid-sensitive PEG chains serving as a pH-responsive polymer layer. The acid-sensitive sheddable PEG moiety undergoes hydrolysis in the acidic tumor microenvironment, exposing mannose ligands to enhance mannose receptor-mediated uptake by TAMs. Both in vitro and in vivo studies demonstrated that this nanosystem improved tumor targeting, resulting in significant tumor growth inhibition and reduced systemic toxicity.

Collectively, these studies demonstrate that pH-responsive polymeric systems have evolved from simple acid-sensitive carriers into multifunctional platforms capable of integrating tumor targeting, intracellular delivery, and combination therapy. Their therapeutic efficacy relies on structural stability during circulation followed by acid-triggered destabilization within the tumor or endosomal environment, resulting in selective drug release and improved intracellular delivery [60]. Despite encouraging preclinical results, heterogeneous tumor acidity, limited predictive animal models, and increasing formulation complexity remain major barriers to clinical translation. Future work should focus on improving pH responsiveness under clinically relevant conditions while simplifying polymer design to enhance reproducibility and translational feasibility.

## 3. Redox-Responsive Polymers

The TNBC microenvironment is characterized by elevated intracellular glutathione (GSH) levels, creating a reductive environment that can be exploited for selective drug release [61,62,63,64]. Redox-responsive polymeric nanocarriers are commonly designed with reduction-sensitive linkages, including disulfide, diselenide, and thioether bonds, which remain stable during circulation but undergo cleavage following intracellular GSH exposure [65]. Bond cleavage subsequently induces polymer degradation, micelle disassembly, or nanoparticle destabilization, resulting in localized drug release within tumor cells while minimizing premature leakage and systemic toxicity [66,67,68]. Owing to their controllable responsiveness and straightforward chemical design, redox-responsive polymers have become one of the most widely investigated platforms for TME-targeted drug delivery in TNBC [69].

**Figure 2:**
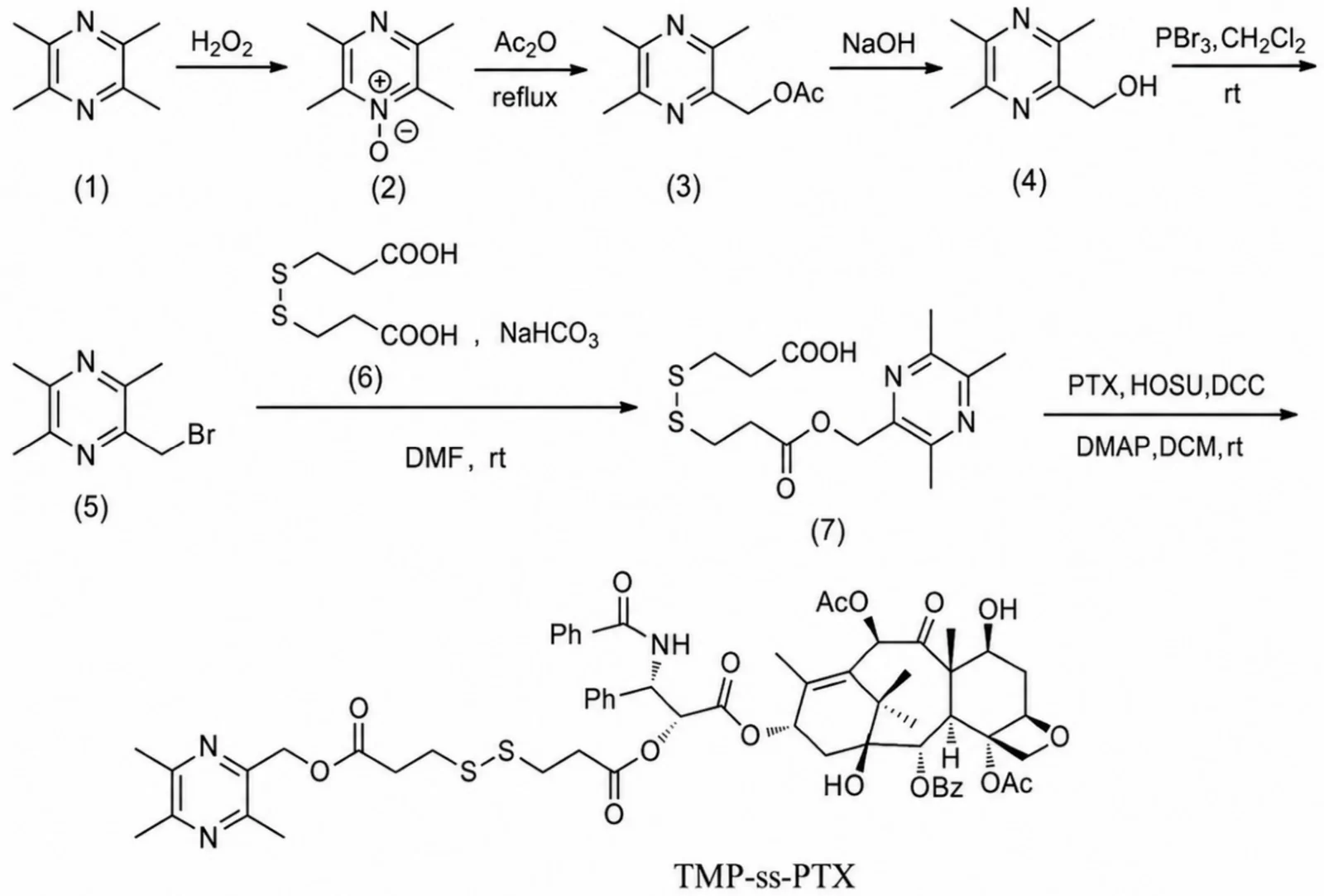
Schematic representation of the synthesis of the PTX-ss-TMP conjugate [70]. Reproduced with Permission, Copyright © 2021, Ivyspring International Publisher.

Zou et al. [70] developed redox-sensitive, carrier-free nanoparticles by linking the chemotherapeutic agent PTX to the anti-angiogenic agent tetramethylpyrazine (TMP) via a disulfide bond. The synthesis of the PTX-ss-TMP conjugate, as illustrated in Figure 2, involves the modification of tetramethylpyrazine through oxidation, acetylation, hydrolysis, and bromination to create a reactive intermediate that is then coupled with 3,3′-dithiodipropionic acid (DTPA) to form the TMP-DTPA conjugate. This intermediate is subsequently reacted with PTX in a condensation reaction using HOSu and DCC as condensing agents and DMAP as a catalyst to yield the final redox-sensitive amphiphilic drug-drug conjugate. This amphiphilic conjugate self-assembles into stable structures that specifically target tumor environments and exploit elevated tumor cell GSH levels for selective drug release. In vitro tests demonstrated that these nanoparticles enhance cellular uptake and increase cancer cell apoptosis compared to traditional combination therapies. Furthermore, the system effectively inhibits angiogenesis in endothelial cells by disrupting the VEGFR2 signaling pathway. In vivo in a mouse model, these nanoparticles exhibited enhanced antitumor efficacy by suppressing tumor growth and prolonging survival [70].

Ma et al. [71] developed redox-responsive poly(oligo(ethylene glycol))-co-poly(vorinostat-disulfide-hyaluronic acid) (POEG-co-PVDSAHA) polymeric micelles for co-delivery of vorinostat (suberoylanilide hydroxamic acid; SAHA), a histone deacetylase inhibitor, and tamoxifen (TAM), a selective estrogen receptor modulator, to sensitize TNBC to the drug. TNBC normally lack ER, PR, and HER2 expression, rendering conventional hormonal therapy ineffective; SAHA epigenetically reactivates estrogen receptor alpha (ERα), enabling TAM-mediated cytotoxicity. SAHA was conjugated via disulfide linkages to allow selective GSH-triggered release in TME. In vitro, POEG-*co*-PVDSAHA micelles reactivated functional ERα in TNBC cells and exhibited enhanced cytotoxicity compared to free SAHA or TAM. In vivo, intravenous administration of TAM/SAHA-loaded micelles in a 4T1.2 subcutaneous model substantially inhibited tumor growth with minimal systemic toxicity [71].

Yu et al. [72] developed redox-responsive arginine-glycine-aspartic acid (RGD)-targeted polymeric micelles (RPSSD@IR780/docetaxel) for photo-chemotherapy (PCT) of TNBC, co-delivering docetaxel and IR-780 (IR780) photosensitizer to achieve synergistic chemo-phototherapy with tumor-targeted uptake. The micelles were composed of RGD-poly(ethylene glycol)-disulfide-distearoylphosphatidylethanolamine (RGD-PEG-SS-DSPE) polymer, incorporating disulfide bonds that are cleaved in the high GSH in TME, enabling selective intracellular release of docetaxel and IR780. In vitro, drug release studies demonstrated 76.2% docetaxel release under redox conditions (pH 5.5 + 10 mM dithiothreitol [DTT]) over 72 h, compared to 41.2% and 47.4% at neutral and mildly acidic pH, respectively. Cytotoxicity assays in MDA-MB-231 cells showed dose-dependent reduction in cell viability, with maximal apoptosis under near-infrared (NIR) light irradiation. Cellular uptake studies confirmed enhanced internalization mediated by RGD targeting. In vivo, in a subcutaneous MDA-MB-231 tumor model, treatment with RPSSD@IR780/DOC + NIR achieved ~92% tumor suppression, significantly prolonged survival (up to 50 days), reduced proliferation as indicated by Ki67 staining, and increased tumor necrosis, demonstrating the synergistic effect of redox-responsive drug release, RGD targeting, and PCT [72].

Badparvar et al. [73] developed docetaxel (DTX)-loaded dual pH/redox-responsive hyperbranched polymeric nanoparticles (DTX-NPs) for targeted TNBC therapy. The system exhibits pH-responsive charge reversal, switching from a negative charge in the bloodstream to a positive charge in the tumor microenvironment. This behavior is driven by the protonation of tertiary amine groups in the poly(β-amino ester)s (PBAEs) component under mildly acidic conditions (pH~6.4). This charge transition facilitates electrostatic interactions with negatively charged cancer cell membranes, resulting in markedly enhanced cellular uptake. Simultaneously, the redox-responsive behavior is governed by disulfide bonds incorporated into the polymer structure. These bonds are cleaved in response to high intracellular glutathione (GSH) levels, leading to nanoparticle size shrinkage from approximately 80 nm to 32 nm, which may further enhance tumor penetration. In vitro, treatment of MDA-MB-231 cells with DTX-NPs resulted in enhanced cytotoxicity compared with free DTX, accompanied by increased apoptosis and modulation of Bcl-2-associated X protein (Bax) and Bcl-2 expression [73].

Huang et al. [74] developed 2-(diisopropylamino)ethyl methacrylamide-N,N′-disulfide dimethacrylamide-poly(ethylene glycol) microgels (DPA-DSDMA-PEG microgels) as redox- and pH-responsive carriers for DOX delivery in TNBC therapy. The disulfide bonds in the cross-linker (DSDMA) are cleaved by high intracellular GSH, triggering microgel disassembly and redox-controlled DOX release, while the tertiary amine groups in DPA are protonated under acidic conditions (pH < 6.3), increasing hydrophilicity and promoting pH-triggered drug release. In vivo, DOX@DPA-DSDMA-PEG microgels effectively suppressed tumor growth, increased apoptosis, and reduced proliferation, while minimizing systemic toxicity compared to free DOX [74].

Collectively, these studies indicate that redox-responsive polymer systems in TNBC exploit elevated intracellular glutathione as a tumor-selective trigger for controlled drug release. Across disulfide-linked micelles, hyperbranched copolymers, microgels, and dual-responsive nanoparticles, a consistent mechanistic principle is observed: structural stability during circulation, followed by glutathione-mediated bond cleavage after cellular internalization, leading to rapid intracellular payload release. This intracellular activation distinguishes redox-responsive platforms from systems that rely on extracellular triggers and directly leverages a metabolic feature associated with TNBC chemoresistance.

A key development is the integration of redox responsiveness with biologically informed therapeutic strategies. Hyaluronic acid–based micelles co-delivering PTX and betulinic acid combine CD44-mediated targeting with enhanced apoptosis induction [75,76]. Disulfide-linked vorinostat systems introduce an epigenetic dimension by restoring estrogen receptor expression and sensitizing TNBC cells to tamoxifen [77]. RGD-modified micelles enable combined chemo-phototherapy, while dual pH/redox-responsive nanoparticles incorporate charge reversal and size shrinkage to improve cellular uptake and tumor penetration. Together, these approaches extend redox-responsive delivery beyond passive release toward modulation of tumor cell survival pathways and microenvironmental interactions.

Despite encouraging preclinical results, several limitations remain. Intracellular glutathione levels vary across tumor subpopulations, and many in vitro release studies rely on supraphysiological reducing conditions. In addition, most validations are performed in subcutaneous models that do not fully reflect metastatic TNBC. The systemic stability and long-term biodegradation of disulfide-containing polymers also require further investigation.

Overall, redox-responsive polymers provide an effective strategy for exploiting the reductive intracellular environment of TNBC to achieve selective drug release. Future studies should focus on improving polymer performance under clinically relevant redox conditions while simplifying carrier design to enhance reproducibility and translational potential.

## 4. Hypoxia-Responsive Polymers

Hypoxia is a defining feature of TME in TNBC, resulting from rapid tumor growth and insufficient vascularization. Low oxygen levels create a unique biochemical trigger that can be exploited for selective drug delivery, enabling polymers to release therapeutics preferentially in hypoxic tumor regions while minimizing systemic toxicity [78,79,80,81]. Hypoxia-responsive polymers are commonly engineered with bio-reductive linkers, such as azobenzene (AZO) or nitroaromatic groups, which undergo cleavage or structural transformation under low-oxygen conditions. These chemical reactions destabilize the polymeric carrier, inducing micelle or nanoparticle disassembly and releasing encapsulated drugs or nucleic acids specifically in hypoxic zones. Targeting ligands can be combined with hypoxia-responsiveness to enhance selective uptake in hypoxic tumor niches, further improving delivery precision [82]. By aligning drug release with hypoxic regions, these polymers facilitate enhanced intracellular delivery, co-delivery of combination therapies, and suppression of hypoxia-driven tumor progression and metastasis [83,84,85,86,87].

**Figure 3:**
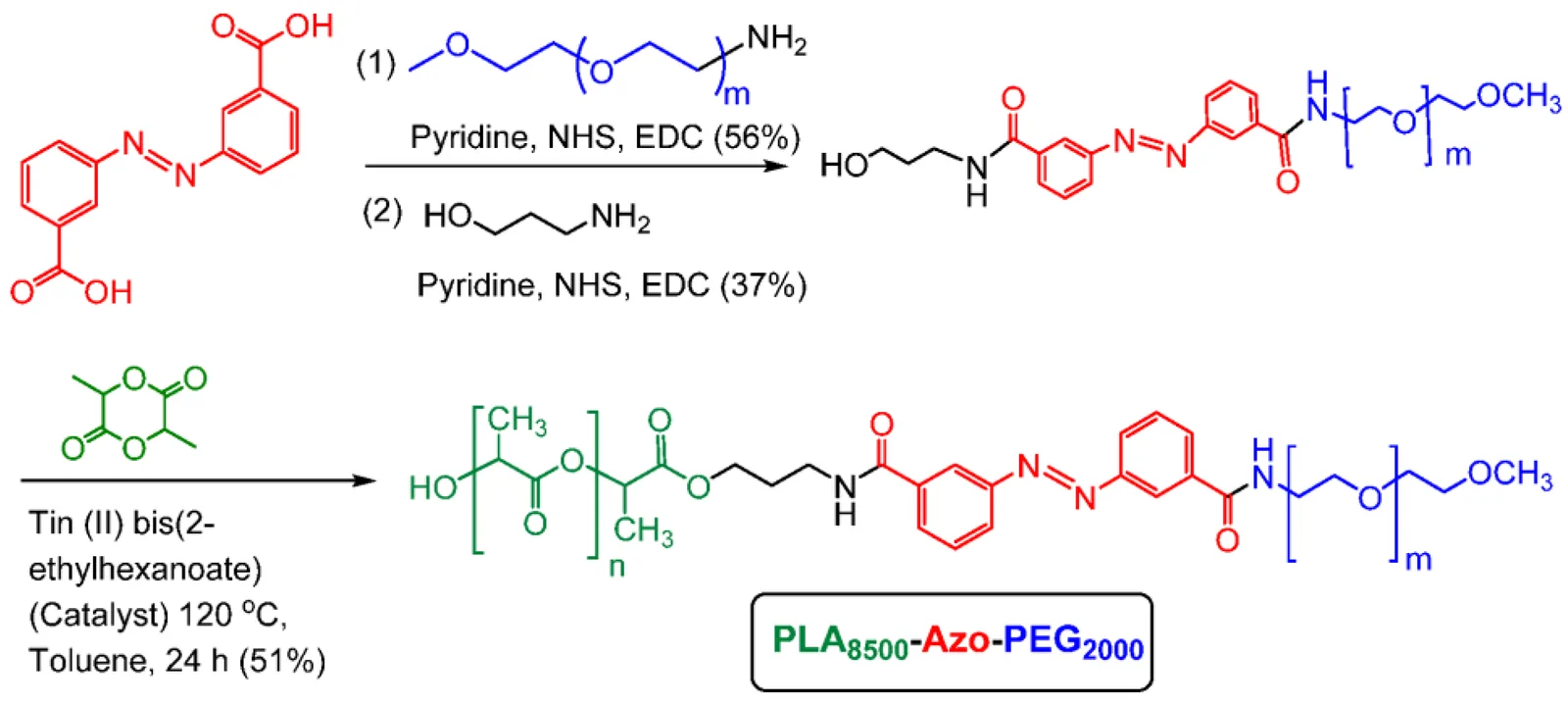
Preparation of the polymer incorporating a hypoxia-sensitive diazobenzene linker [88]. Reproduced with Permission, Copyright © 2021, American Chemical Society.

Mamnoon et al. [88] developed hypoxia-responsive, iRGD-targeted DOX-loaded polymeric nanoparticles (DOX-iPs) for TNBC treatment. These nanoparticles were synthesized from the diblock copolymer composed of poly(lactic acid) and poly(ethylene glycol) bridged through a diazobenzene linker (PLA-diazobenzene-PEG), in which the diazobenzene linker is reduced under hypoxic conditions, enabling selective DOX release in low-oxygen tumor regions (Figure 3). In vitro, targeted polymersomes exhibited enhanced cellular uptake and cytotoxicity under hypoxia compared with non-targeted vesicles or free DOX, consistent with iRGD-mediated Neuropilin-1 receptor targeting. In vivo, DOX-iPs significantly suppressed tumor growth and minimized off-target toxicity, demonstrating efficient, tumor-specific drug delivery and improved antitumor efficacy [88].

Lu et al. [89] developed hypoxia-responsive stereo-complex polymeric micelles (AZO-SC-PMs/DTX) for targeted delivery of DTX and inhibition of breast cancer metastasis. The micelles were synthesized from enantiomeric poly(D-lactide) (PDLA) and poly(L-lactide) (PLLA) combined with methylated PEG (mPEG), incorporating AZO moieties that undergo reductive cleavage under hypoxic conditions, triggering drug release specifically in low-oxygen tumor regions. In vitro, these micelles enhanced cellular uptake, cytotoxicity, and inhibition of cell migration under low-oxygen conditions compared with non-responsive micelles or free DTX. In vivo, AZO-SC-PMs/DTX markedly suppressed tumor growth in orthotopic 4T1 models, increased tumor necrosis, and reduced metastasis to the lungs and liver, demonstrating that hypoxia-triggered stereo-complex micelles can improve antitumor efficacy and inhibit tumor spread while maintaining tumor specificity [89].

Liu et al. [90] developed hypoxia-responsive polymeric micelles (AZO-PMs) co-delivering DOX and short hairpin RNA targeting HIF-1α (shHIF-1α) for the treatment of TNBC. The micelles were synthesized from methoxy-polyethylene glycol (mPEG) and poly-L-lysine (PLL) with an AZO linker that undergoes reductive cleavage under hypoxic conditions, triggering the disassembly of the micelles and release of therapeutic agents. In vitro, AZO-PMs enhanced DOX release and efficiently delivered shHIF-1α, achieving greater HIF-1α silencing, reduced cell viability, and inhibited migration compared with non-responsive micelles. In vivo, orthotopic MDA-MB-231 tumors treated with AZO-PMs/DOX + shHIF-1α exhibited markedly inhibited tumor growth, increased tumor necrosis, decreased proliferation, and reduced lung metastasis, demonstrating the enhanced antitumor and anti-metastatic efficacy of this hypoxia-responsive co-delivery system [90].

Weng et al. [91] developed hypoxia-responsive polymeric micelles (AZO-PLA) co-delivering DOX and digoxin (DIG), a HIF-1α inhibitor used to suppress hypoxia-induced drug resistance, for targeted treatment of TNBC. The hypoxia responsiveness of this system is governed by an AZO linker that connects the hydrophilic mPEG and hydrophobic PLA segments. Under hypoxic conditions, this AZO bond undergoes bioreductive cleavage by intracellular reductases, leading to micelle disassembly and site-specific drug release. In vitro studies in MDA-MB-231 cells showed that the AZO-PLA micelles released more than 70% of DOX within 24 h under hypoxia, compared with less than 30% from non-responsive micelles. Co-delivery of DOX and DIG resulted in strong synergistic cytotoxicity (optimal molar ratio 3.44/0.05, CI = 0.31), with a marked reduction in cell viability in both monolayer and spheroid cultures. Gene and protein analyses further confirmed DIG-mediated inhibition of HIF-1α, VEGF, and MMP9 under hypoxic conditions, as demonstrated by RT-qPCR, immunofluorescence, and Western blotting. In addition, wound healing assays showed that AZO-PLA/DOX+DIG effectively suppressed cell migration. In vivo, treatment of orthotopic MDA-MB-231 tumors with AZO-PLA/DOX+DIG resulted in the greatest tumor growth inhibition compared with free DOX, PLA/DOX, and AZO-PLA/DOX. This formulation also reduced tumor volume and weight and suppressed lung metastasis, supporting the therapeutic potential of hypoxia-responsive co-delivery of DOX and DIG [91].

Edvall et al. [92] developed hypoxia-responsive polymersomes based on PLA and mPEG linked through a diazobenzene moiety for co-delivery of DOX and all-trans retinoic acid (ATRA), a differentiation agent that reduces cancer stem cell stemness. The diazobenzene linker is reduced under hypoxic conditions, triggering drug release in hypoxic tumor regions. In TNBC cells, the DOX + ATRA combination enhanced cellular uptake and showed improved cytotoxicity under hypoxia compared with DOX alone. The treatment also reduced cell migration and significantly decreased spheroid growth, indicating inhibition of invasive and stem-like tumor phenotypes. In vivo, hypoxia-responsive polymersomes further improved tumor growth inhibition in patient-derived xenograft models relative to DOX alone, supporting the potential of this system for targeting aggressive TNBC [92].

Collectively, these studies demonstrate that hypoxia-responsive polymeric systems provide an effective strategy for selective drug activation in TNBC by exploiting low-oxygen regions within tumors. Across different polymer designs, including azobenzene- and diazobenzene-linked systems, hypoxia-induced bond cleavage destabilizes the carrier and enables localized drug release. Recent advances have further integrated hypoxia responsiveness with biologically informed payloads, such as digoxin, shHIF-1α, and ATRA, to suppress HIF-1α signaling, reduce cancer stemness, and overcome hypoxia-associated therapeutic resistance. Targeting ligands, including RGD and iRGD, further enhance accumulation within hypoxic tumor regions.

Despite these advances, several limitations remain. Tumor hypoxia is spatially and temporally heterogeneous, which may result in inconsistent activation of hypoxia-responsive systems. Moreover, most studies rely on simplified hypoxia models or murine tumor models that do not fully recapitulate metastatic TNBC, while polymer stability, systemic safety, and scalable manufacturing remain important translational challenges. Future studies should focus on optimizing hypoxia sensitivity under clinically relevant conditions, validating efficacy in representative tumor models, and simplifying polymer design to facilitate clinical translation.

## 5. ROS-Responsive Polymers

TME of TNBC is characterized by high levels of reactive oxygen species (ROS), including hydrogen peroxide, superoxide, and hydroxyl radicals, arising from mitochondrial dysfunction, accelerated metabolism, and oncogenic signaling, which contribute to tumor progression, invasion, and therapy resistance [93,94,95]. These pathological ROS levels provide a unique trigger for ROS-responsive polymeric systems, which are engineered with ROS-sensitive linkages, such as thioketal, boronic ester, or peroxalate bonds, that remain stable in normal tissues but undergo selective cleavage in the ROS-rich TME, leading to controlled degradation of the polymer backbone and localized release of encapsulated therapeutics [96]. By responding dynamically to the oxidative gradients within tumors, these polymers facilitate enhanced tumor penetration, uniform drug distribution, and site-specific therapy, thereby improving therapeutic efficacy while minimizing systemic toxicity [97,98,99,100].

Zhang et al. [101] developed ROS-responsive Fenton-reaction-stimulative nanoparticles (P@P/H NPs) composed of PLGA and low-molecular-weight polyethyleneimine (PEI), incorporating a β-cyclodextrin–ferrocene molecular switch (B-CD@Fc) to enhance DOX therapy against TNBC. In hydrogen peroxide (H_2_O_2_)-rich TME, the B-CD@Fc complex dissociates, triggering ferrocene-mediated Fenton reactions and excessive hydroxyl radical generation, which amplifies intracellular ROS, promotes apoptosis, and suppresses metastasis. In vitro studies using 4T1 cells demonstrated that P@P/H nanoparticles (NPs) exhibit significantly enhanced antitumor efficacy compared with free DOX. P@P/H NPs showed dose-dependent cytotoxicity with a markedly lower IC_50_ (0.50 ± 0.02 µg/mL) than free DOX (2.04 ± 0.31 µg/mL), indicating improved therapeutic potency. Clonogenic assays confirmed strong anti-proliferative effects, reducing colony formation from 38% in controls to 6% after treatment. Confocal microscopy and flow cytometry revealed substantially enhanced cellular uptake of DOX when delivered via P@P/H NPs. Additionally, P@P/H NPs elevated intracellular ROS levels and significantly inhibited cell migration, as shown by wound healing assays. Western blot analysis further demonstrated activation of mitochondrial apoptosis pathways and suppression of metastasis-related matrix metalloproteinase-9 (MMP-9) expression. In vivo, in a 4T1 xenograft mouse model, P@P/H NPs showed the strongest tumor growth inhibition, yielding a lower tumor weight (0.42 ± 0.13 g) and higher tumor inhibition rate (56.37%) compared with free DOX (0.97 ± 0.08 g, 37.54%). Notably, body weight remained stable in the P@P/H NP group, whereas free DOX induced weight loss [101].

**Figure 4:**
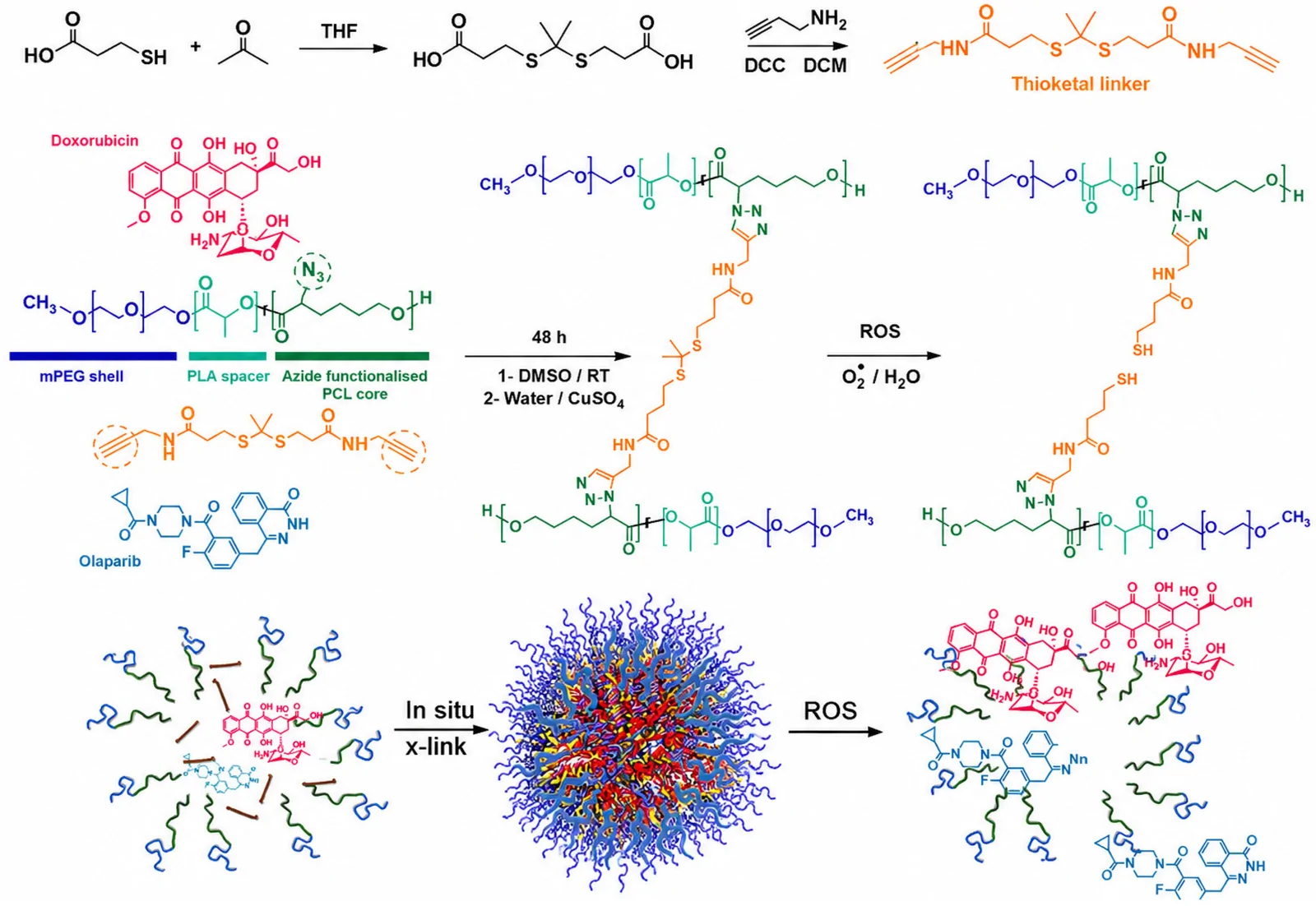
Illustration of the synthesis of ROS-responsive nanoparticles, in which a bis(alkynyl) thioketal cross-linker is reacted with mPEG-b-poly(D,L-lactide-co-azido-caprolactone) in the presence of DOX and OLA to produce drug-loaded nanoparticles capable of releasing their payload selectively in reactive oxygen species (ROS)-rich environments [102]. Reproduced from Ref. [102], under the terms of the CC BY 3.0 license.

Cavanagh et al. [102] explored the co-delivery of DOX and olaparib (OLA), a PARP inhibitor, using ROS-responsive polymeric nanoparticles to achieve synergistic antitumor effects. These nanoparticles were designed as micellar constructs composed of PEG for colloidal stability and poly(D,L-lactide-co-2-azidocaprolactone) (PLA-azide) to enable core cross-linking via click chemistry, with an oxidation-sensitive dithioketal (DTK) linker incorporated to confer ROS-triggered degradability. The polymer was synthesized through ring-opening copolymerization of D,L-lactide and chloro-ε-caprolactone with mPEG, followed by azide functionalization of the pendant caprolactone units. Drug loading was achieved during the cross-linking step with the DTK linker, forming micellar nanoparticles with a hydrophilic PEG corona and ROS-sensitive core. In the ROS-rich TME, the dithioketal bonds are cleaved, triggering nanoparticle disassembly and controlled, tumor-selective release of DOX and OLA, providing a platform for enhanced combination therapy and potential integration with radiotherapy interventions (Figure 4). In vitro studies demonstrated that TNBC cell lines MDA-MB-231 and MDA-MB-468 exhibited relative resistance to DOX alone, as indicated by higher IC_50_ values compared to the luminal breast cancer line MCF-7. Co-delivery of DOX and OLA at ratios of 100:1, 10:1, and 1:1 produced strong synergistic cytotoxicity (CI < 0.9) in TNBC models, while higher OLA ratios triggered antagonism. These ratio-dependent results were validated in both 2D and 3D spheroid models. Furthermore, their ROS-responsive nanoparticles significantly improved therapeutic outcomes by enhancing cellular uptake and nuclear localization of DOX, leading to greater reductions in metabolic activity and clonogenic survival compared to free drugs [102].

Li et al. [103] developed curcumin-loaded biotin-functionalized nanoparticles (CUR@Bio/PE-NPs) as ROS-responsive nanocarriers for targeted TNBC therapy. The system incorporates peroxalate ester linkages that are cleaved by H_2_O_2_ in ROS-rich tumor environments, triggering nanoparticle disassembly and curcumin release. Notably, curcumin further elevates intracellular ROS levels, creating a positive feedback mechanism that enhances drug release. In TNBC cells, CUR@Bio/PE-NPs showed increased cellular uptake, enhanced ROS generation, and improved cytotoxicity compared with non-targeted formulations. The nanoparticles also promoted apoptosis, induced G2/M cell-cycle arrest, and inhibited cell migration and invasion. In vivo, CUR@Bio/PE-NPs exhibited preferential tumor accumulation and achieved greater tumor growth inhibition than control formulations in a 4T1 model, while maintaining good safety profiles. In addition, lung metastasis was significantly reduced, supporting the therapeutic potential of ROS-responsive, biotin-targeted nanoparticles for TNBC [103].

ROS-responsive polymeric nanocarriers incorporating phenylboronic acid or boronic ester linkages have been widely explored as a strategy for TNBC therapy [104]. Zhou et al. [105] developed DOX-loaded, galactose-functionalized polyacrylate nanoparticles (DOX@NPs) for ROS-responsive drug delivery. The system combines a galactose-modified polymer for targeting with a phenylboronic acid–containing polymer that enables ROS-triggered DOX release. In ROS-rich tumor environments, boronic ester bonds are cleaved, leading to nanoparticle disassembly and controlled intracellular drug release. In TNBC cells, DOX@NPs showed higher cellular uptake and improved cytotoxicity compared with free DOX. Apoptosis and cell-cycle analyses indicated increased apoptosis and G2/M phase arrest following treatment. In vivo, DOX@NPs preferentially accumulated in tumor tissue and achieved greater tumor suppression than free DOX, while showing minimal systemic toxicity, supporting the potential of ROS-responsive nanoparticles for targeted TNBC therapy [105].

Collectively, ROS-responsive polymeric systems exploit elevated ROS levels in the TNBC microenvironment to achieve selective intracellular drug release. These carriers remain stable under physiological conditions but undergo ROS-mediated cleavage within tumor cells, resulting in controlled drug release and enhanced therapeutic efficacy. Recent advances have extended these systems beyond ROS-triggered delivery by incorporating ROS-amplifying strategies, such as Fenton or Fenton-like reactions, and co-delivering complementary therapeutic agents within ROS-cleavable matrices, thereby improving tumor penetration and therapeutic synergy [96]. Functionalization with targeting ligands, including biotin and galactose, further enhances cellular uptake and tumor selectivity.

Despite these advances, several limitations remain. Intratumoral ROS levels are spatially and temporally heterogeneous, which may lead to inconsistent activation of ROS-responsive carriers, while elevated ROS in inflammatory tissues raises the possibility of off-target activation [96]. Moreover, most studies have been validated in subcutaneous xenograft models, and the long-term stability, degradation behavior, pharmacokinetics, systemic safety, and scalable manufacture of these systems remain insufficiently investigated. Future studies should focus on optimizing ROS responsiveness under clinically relevant conditions, validating performance in representative tumor models, and simplifying polymer design while integrating complementary TME-responsive mechanisms to facilitate clinical translation.

## 6. Photo-Responsive Polymers

Photo-responsive polymeric systems are a class of smart nanocarriers that respond to light stimuli to achieve controlled drug delivery. These systems incorporate photo-sensitive moieties, such as azobenzene, spiropyran, or conjugated polymers, which undergo structural or chemical changes upon exposure to specific wavelengths of light [106,107]. This photo-induced transformation can trigger drug release, modulate carrier properties, or generate ROS for photodynamic therapy (PDT). PDT is a therapeutic approach in which a photosensitizer is activated by light to generate ROS, thereby inducing oxidative damage and tumor cell death. The unique feature of photo-responsive systems is their spatiotemporal precision, allowing selective activation of therapeutic agents at the tumor site while minimizing damage to surrounding healthy tissues. By integrating these systems with targeting ligands or co-delivery platforms, it is possible to combine chemotherapy with phototherapy, offering a versatile strategy for enhanced treatment of aggressive cancers, including triple-negative breast cancer [108,109,110,111].

**Figure 5:**
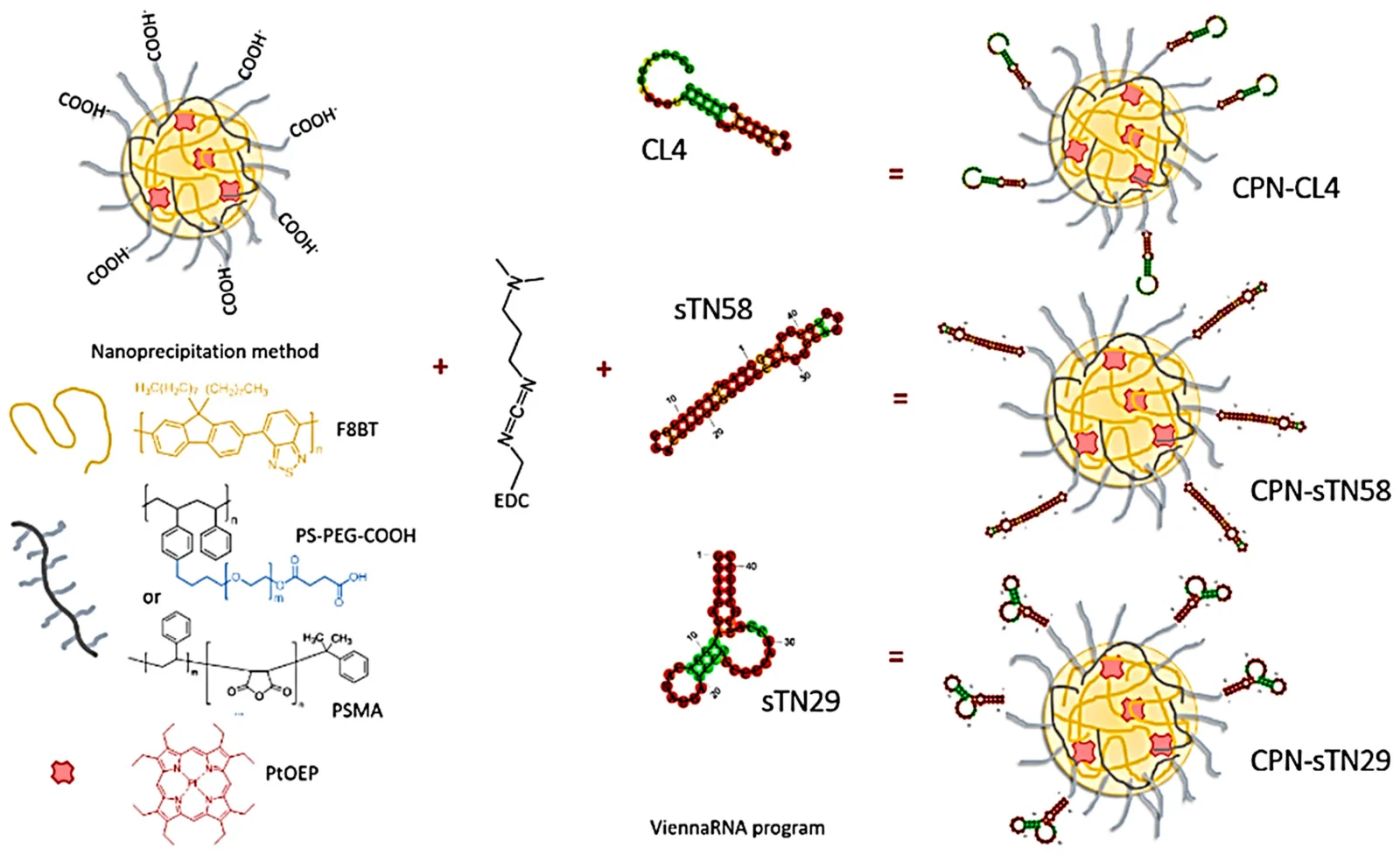
Functionalization of metallated porphyrin-incorporated conjugated polymer nanoparticles (CPNs) with aptamers via EDC-mediated coupling chemistry for targeted photodynamic treatment of TNBC. Reproduced from Ref. [112], under the terms of the CC BY 4.0 license.

Ibarra et al. [112] developed conjugated polymer nanoparticles (CPNs) functionalized with RNA aptamers for selective PDT against TNBC. The nanoparticles, composed of poly(9,9-dioctylfluorene-alt-benzothiadiazole) (F8BT) and polystyrene-based polymers (PS-PEG-COOH and PSMA), generate ROS upon light activation, disrupting cancer cell redox balance and inducing apoptosis. Figure 5 illustrates the decoration of metallated porphyrin-doped CPNs with aptamers via EDC coupling. The fluorescent polymer (F8BT) transfers energy to Pt(II) octaethylporphyrin (PtOEP), while carboxyl groups on PS-PEG-COOH and PSMA are activated by EDC to link NH_2_-modified aptamers (CL4, sTN58, sTN29), enhancing targeting, cellular uptake, and PDT efficacy in TNBC cells. In vitro studies evaluated aptamer-decorated conjugated polymer nanoparticles (CPNs) for PDT against TNBC cells. Flow cytometry and confocal imaging demonstrated that CPNs functionalized with specific RNA aptamers, such as CL4, sTN58, and sTN29, exhibited markedly higher selective uptake and effective intracellular localization in MDA-MB-231 and BT-549 cells compared to non-decorated or scrambled controls. Dark cytotoxicity assays confirmed the formulation’s biocompatibility, with cell viability remaining above 90% at concentrations up to 10 mg/L. Upon light activation, the targeted PDT significantly reduced cell viability; specifically, sTN58-decorated CPNs were able to lower viability to approximately 24% and induce 61% apoptosis in chemo-resistant MDA-MB-231 cells. TNBC xenograft mouse models demonstrated that the aptamer-functionalized CPNs preferentially accumulated in tumor tissue, enhancing the therapeutic efficacy of PDT and leading to significant tumor growth inhibition compared to controls. No major systemic toxicity was observed, indicating the biocompatibility of the formulation [112].

Patra et al. developed [113] a highly efficient photo-switchable smart polymeric nano-vehicle for targeted gene and drug delivery in TNBC. The polymeric system was synthesized using a photo-switchable spiropyran acrylate (SPA) moiety and an amino-acid-based cationic segment, polyhydroxyethyl methacrylate-conjugated phenylalanine (P(HEMA-Phe-NH_2_)), through RAFT polymerization, allowing the nano-vehicle to respond to light stimuli. The system leverages elevated Cu^2+^ levels in TNBC for targeted delivery and diagnosis. The polymer exhibits photo-responsiveness via reversible isomerization between the hydrophobic spiropyran (SP) form and the hydrophilic merocyanine (MC) form, induced by UV light (365 nm) and reversed by visible light (520 nm). This conversion triggers structural changes that enable the controlled release of encapsulated hydrophobic drug DOX. In vitro studies evaluated a DOX-loaded P(SPA-co-HEMA-Phe-NH_2_) polymeric nanovehicle in MDA-MB-231 cells. The polymer exhibited low toxicity alone, while UV-triggered DOX release significantly enhanced cytotoxicity (IC_50_ = 1.15 µg/mL vs. 4.6 µg/mL under visible light). Fluorescence imaging confirmed efficient nuclear uptake of DOX under UV activation. JC-1 (5,5′,6,6′-Tetrachloro-1,1′,3,3′-tetraethylbenzimidazolylcarbocyanine iodide) assays showed mitochondrial depolarization, and DCFDA assays revealed increased ROS generation, indicating effective apoptosis induction. These results highlight the polymer’s potential as a light-activated anticancer drug delivery system [113].

Xiang et al. [114] developed a photosensitive smart hydrogel loaded with neratinib (NB) for NIR-triggered PDT to improve TNBC treatment outcomes. The hydrogel was synthesized using Methacrylate hydroxybutyl chitosan (MHBC) for structural integrity, Poly(N-isopropyl acrylamide) (PNIPAM) for thermoresponsiveness, and AZO as a photoresponsive component. The AZO moiety undergoes reversible trans–cis isomerization under light exposure, which changes the hydrogel’s physical properties and enables controlled drug release. In vitro studies evaluated a photosensitive smart hydrogel loaded with neratinib (NB) against MDA-MB-231 cells. The NIR-activated formulation (M/PP/A/NB+NIR) showed the highest cytotoxicity in MTT assays, with AO/EB staining confirming extensive apoptosis. ROS measurements indicated significant reactive oxygen species generation, while JC-1 staining revealed mitochondrial depolarization, supporting apoptosis induction. Reverse Transcription Polymerase Chain Reaction (RT-PCR) analysis showed upregulation of p53 and downregulation of Mitogen-Activated Protein Kinase (MAPK) pathway genes, highlighting the hydrogel’s ability to target key cancer survival pathways. These results demonstrate that the NB-loaded, NIR-responsive hydrogel effectively enhances cytotoxicity and pro-apoptotic activity in TNBCs. In vivo studies using a 4T1 xenograft model showed that the NIR-activated hydrogel (M/PP/A/NB+NIR) markedly inhibited tumor growth. This treatment significantly reduced tumor volume and weight compared to controls, and histopathology revealed decreased tumor cell density with extensive necrosis [114].

**Figure 6:**
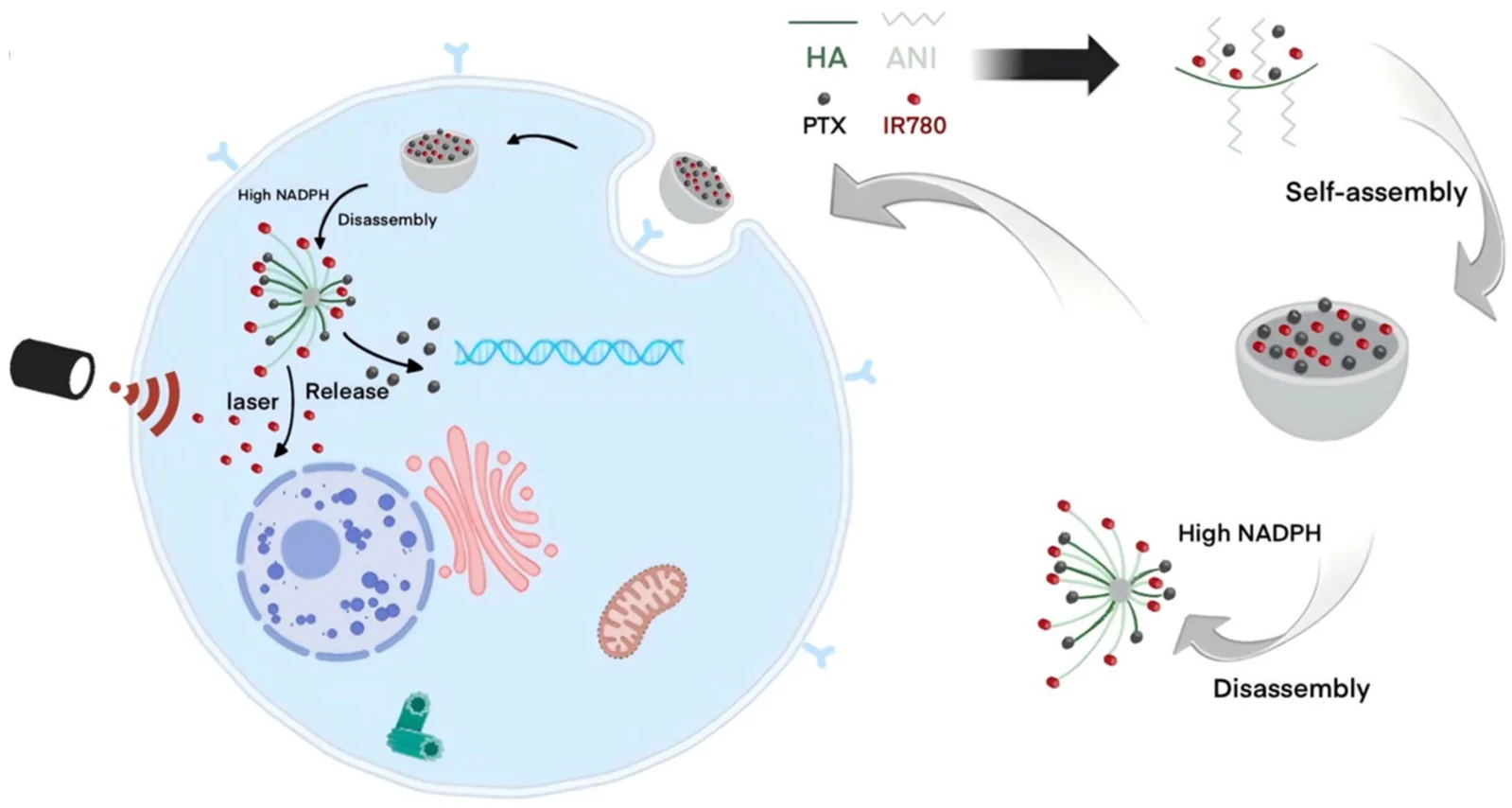
Schematic illustration of the P/I nanoparticle preparation and their subsequent behavior during combination therapy, including cellular uptake and drug release triggered by the tumor microenvironment [115]. Reproduced from Ref. [115], under the terms of the CC BY-NC 3.0 license.

Yang et al. [115] developed PTX- and IR780-co-loaded nanoparticles (P/I NPs) based on the amphiphilic polymer HA-ANI, in which hyaluronic acid (HA) was grafted with 2-nitroimidazole (ANI). The ANI moiety confers hypoxia responsiveness by promoting nanoparticle destabilization under hypoxic conditions, whereas the encapsulated IR780 enables NIR-triggered photothermal therapy. This dual-responsive design combines hypoxia-triggered drug release with light-mediated therapeutic activation, thereby enhancing antitumor efficacy. Figure 6 illustrates the photoresponsive behavior by showing how an external NIR laser interacts with the internalized nanoparticles to trigger drug release and activate phototherapy. This process is driven by the encapsulated IR780 dye, which converts the laser’s energy into heat to destroy tumor cells, a mechanism that remains stable thanks to the protective HA-ANI polymer shell. In vitro assays on 4T1 cells revealed that these nanoparticles achieve a high cellular uptake rate of 91.50% via CD44 receptor-mediated endocytosis, leading to significantly enhanced cytotoxicity and reduced cell viability compared to free drugs. The formulation effectively suppressed cancer cell migration to just 2.68% and induced higher apoptosis rates through the synergistic effects of chemotherapy and NIR laser-triggered phototherapy. In vivo testing using 4T1 xenograft models demonstrated potent antitumor efficacy, with P/I NPs reducing tumor weight by 59.95% and achieving a growth inhibition rate of 35.44%. Finally, histopathological analysis confirmed extensive tumor necrosis, while H&E staining of major organs indicated excellent biocompatibility with no significant side effects [115].

Collectively, photo-responsive polymeric systems provide precise spatiotemporal control over therapeutic activation while integrating targeted drug delivery with phototherapy. Recent advances, including aptamer-functionalized conjugated polymer nanoparticles, photo-switchable polymeric nanovehicles, and NIR-responsive hydrogels, have improved tumor selectivity, controlled drug release, and therapeutic efficacy in TNBC models [116,117]. These platforms also enable combination strategies that couple photoactivation with chemotherapy or photodynamic therapy to enhance antitumor activity.

Despite these advances, several challenges remain. Limited light penetration, particularly for UV and visible wavelengths, restricts treatment of deep-seated tumors, while dependence on external irradiation may reduce clinical applicability and increase the risk of off-target phototoxicity [118,119]. In addition, the structural complexity of many photo-responsive systems, together with concerns regarding polymer biodegradability, long-term safety, and scalable manufacture, continues to hinder clinical translation. Future studies should focus on improving tissue penetration through NIR-responsive systems, simplifying polymer design, and integrating complementary TME-responsive mechanisms to enhance therapeutic precision and translational potential [120,121].

## 7. Temperature-Responsive Polymers

Temperature-responsive polymers exploit the mildly elevated tumor temperatures or externally applied thermal stimuli in TNBC to achieve site-specific drug release. Such polymers are designed to undergo a reversible phase transition at their lower critical solution temperature (LCST), typically between 37~45 °C, enabling a hydrophilic-to-hydrophobic shift that triggers nanoparticle collapse and controlled release of encapsulated therapeutics. Thermo-responsive polymers respond to temperature changes by altering their solubility, conformation, or membrane interaction, which enhances tumor penetration and cellular uptake. By aligning therapeutic release with tumor-specific thermal cues, temperature-responsive polymers facilitate enhanced intracellular delivery, combination therapy integration, and synergistic treatment efficacy [122,123,124,125].

Ribeiro et al. [126] developed thermo-responsive nanoparticles composed of N-isopropylacrylamide (NIPAM) copolymerized with poly(ethylene glycol) methacrylate (PEGMA) and allylamine (AA) for targeted delivery of DOX in TNBC. Nanoparticles were designed to exploit elevated tumor temperatures to trigger DOX release via LCST behavior of PNIPAM, while folic acid functionalization enhanced tumor targeting. In vitro, 2D monolayer assays with L929 fibroblasts showed high biocompatibility, and MDA-MB-468 TNBC cells exhibited improved cytotoxicity with folic acid-functionalized NPs. In 3D spheroid models generated from MDA-MB-468 TNBC cells, the targeted NPs effectively penetrated the spheroids and reduced cell viability in a temperature-dependent manner [126].

Kurowska et al. [127] developed membrane-active, thermo-responsive block copolymers containing poly(N-isopropylacrylamide) (PNIPAAm) and poly(N-vinylcaprolactam) (PNVCL) for targeted delivery of DOX in TNBC therapy. Those segments are the specific components responsible for the polymer’s thermoresponsive behavior. These hydrophilic blocks exhibit an LCST, which enables the copolymer to undergo a sharp phase transition and self-organize in response to temperature changes, enabling encapsulation and controlled release of hydrophobic drugs such as DOX. In vitro, DOX-loaded nanoparticles significantly reduced the viability of MDA-MB-231 cells, inducing higher levels of apoptosis and necrosis compared with free DOX. Hemocompatibility tests confirmed the polymers were non-hemolytic, demonstrating good biocompatibility [127].

Zhao et al. [128] developed a thermosensitive hydrogel system co-loading PTX nanocrystals and niclosamide (NLM) nanocrystals (PN-NCs-Ts Gel) stabilized within a poly(lactic-co-glycolic acid)-poly(ethylene glycol)-poly(lactic-co-glycolic acid) (PLGA-PEG-PLGA) hydrogel for intratumoral treatment of TNBC, providing sustained drug release over several days. The hydrogel exhibits thermosensitive behavior via reversible gel-sol transitions of the triblock copolymer, enabling controlled release of PTX and NLM nanocrystals. In vitro, PN-NCs-Ts Gel exhibited synergistic cytotoxicity in MDA-MB-231 TNBC cells, induced enhanced apoptosis, and significantly inhibited cell migration. In MDA-MB-231 xenograft-bearing mice, PN-NCs-Ts Gel efficiently suppressed tumor growth compared with individual treatments, maintained stable body weight, and showed minimal systemic toxicity. Histological analysis revealed extensive tumor necrosis, reduced Ki-67 expression, and a decreased population of breast cancer stem cells (BCSCs), demonstrating effective targeting of both bulk tumor cells and BCSCs [128].

Ding et al. [129] developed thermo-responsive polymer-dopamine core-shell nanostructures (NP-DTS-PDA) composed of poly(2-(2-methoxyethoxy)ethyl methacrylate-co-oligo(ethylene glycol) methyl ether methacrylate-co-2-(dimethylamino)ethyl methacrylate)-b-poly(lactic-co-glycolic acid) (P(MEO2MA-co-OEGMA-co-DMAEMA)-b-PLGA)) for targeted therapy of TNBC. The nanoparticles exhibit an LCST, enabling temperature-induced nanoparticle collapse and controlled release of DOX. In addition, the polydopamine (PDA) shell provides photothermal responsiveness, converting localized NIR light into heat, which can further enhance drug release. In vitro, NP-DTS-PDA significantly reduced the viability of MDA-MB-231 TNBC cells, inducing approximately 80% tumor cell death. In vivo, in an orthotopic TNBC model, NP-DTS-PDA treatment achieved marked tumor regression with extensive apoptosis and necrosis, while maintaining normal liver, heart, and kidney function, indicating minimal systemic toxicity [129].

Bobrin et al. [130] developed thermoresponsive polymeric tadpole nanostructures coated with PNIPAM for targeted delivery of DOX to TNBC cells. The PNIPAM coating exhibits an LCST, enabling a hydrophilic-to-hydrophobic transition that enhances cellular uptake in tumor environments. In vitro, tadpole nanoparticles showed nearly complete internalization in MDA-MB-231 cells, approximately 300-fold higher than spherical nanoparticles, primarily via phagocytosis. In vivo, biodistribution studies in a subcutaneous MDA-MB-231 mouse model demonstrated selective accumulation at the tumor site, indicating efficient and targeted DOX delivery with minimal off-target effects [130].

Collectively, temperature-responsive polymeric systems provide a versatile approach for TNBC therapy by enabling site-specific drug release in response to endogenous or externally applied thermal stimuli. Recent advances include thermo-responsive nanoparticles and hydrogels with tunable LCST values, as well as multifunctional platforms that combine temperature responsiveness with photothermal therapy or active targeting, resulting in improved drug delivery and antitumor efficacy (Table 1) [131,132,133,134].

Despite these advances, several challenges remain. The relatively small temperature difference between tumors and normal tissues, together with heterogeneous heat distribution and limited penetration of external thermal stimuli, may compromise treatment precision. In addition, the structural complexity, long-term biocompatibility, and scalable manufacture of some temperature-responsive polymers remain important barriers to clinical translation. Future studies should focus on optimizing LCST under physiologically relevant conditions, simplifying polymer design, and integrating complementary TME-responsive mechanisms to improve therapeutic precision and translational feasibility.

## 8. Polymer Design Considerations for Clinical Translation

### 8.1. Polymer Biocompatibility and Biodegradability

Beyond responsiveness to tumor-associated stimuli, the intrinsic properties of polymeric materials largely determine whether a nanocarrier can achieve meaningful therapeutic benefit in vivo. Biocompatibility is particularly important because polymeric nanoparticles inevitably interact with plasma proteins, immune cells, vascular endothelium, and healthy tissues after systemic administration [135,136]. These interactions influence protein corona formation, circulation time, biodistribution, and immune recognition, ultimately affecting therapeutic efficacy. Consequently, polymers with established biocompatibility, including PEG, PLA, PLGA, PCL, and HA, continue to dominate clinically relevant nanocarrier design [137,138,139]. In contrast, highly cationic polymers such as PEI and PDMAEMA often provide superior nucleic acid delivery but may induce membrane damage, oxidative stress, and complement activation in a dose-dependent manner [140]. Future polymer design should therefore balance delivery efficiency with acceptable biosafety rather than maximizing responsiveness alone.

Biodegradability is equally important for clinical translation. Biodegradable polymers are metabolized through established physiological pathways, reducing the risk of long-term accumulation following repeated administration. For example, PLA and PLGA degrade into lactic acid and glycolic acid, whereas HA is enzymatically degraded into low-toxicity oligosaccharides [141]. However, degradation should not simply be rapid. Excessively fast degradation may compromise carrier integrity and drug retention, whereas overly slow degradation may prolong polymer persistence in vivo. Matching degradation kinetics to the therapeutic window therefore remains a key consideration in polymer design.

### 8.2. Degradation Behavior and Degradation-Product Toxicity

Polymer degradation not only governs drug release but also influences the biological response to the carrier itself. Polyester-based polymers generally undergo hydrolytic degradation, whereas enzyme-responsive polymers can achieve more localized degradation within the TME [141]. Importantly, degradation products are not always biologically inert. Local accumulation of acidic degradation products from PLA or PLGA may alter the surrounding microenvironment and affect both tissue responses and the stability of acid-sensitive therapeutics [142]. Likewise, degradation of cationic polymers may generate positively charged fragments that retain membrane-disruptive activity. These considerations highlight that degradation behavior should be evaluated not only in terms of release kinetics but also with respect to degradation-product safety and biological compatibility.

### 8.3. Molecular Weight and Physicochemical Properties

Polymer molecular weight and other physicochemical properties have a substantial influence on nanocarrier performance. Increasing molecular weight generally improves structural integrity and prolongs circulation, which may enhance passive tumor accumulation. However, excessive molecular weight may reduce biodegradation and clearance, increasing the likelihood of tissue retention [135,143]. Surface characteristics, including particle size, surface charge, hydrophobicity, and PEG density, further determine protein adsorption, macrophage uptake, cellular internalization, and biodistribution [135,136]. Although positively charged nanoparticles often exhibit improved cellular uptake, they also show greater nonspecific interactions with biological membranes and serum proteins. Consequently, optimizing physicochemical properties requires balancing delivery efficiency with systemic safety rather than maximizing any single parameter.

### 8.4. Structural Stability and Clinical Translation

Structural stability remains essential for successful clinical translation. Many polymeric micelles undergo premature dissociation after intravenous administration because dilution below the critical micelle concentration can result in early drug leakage and reduced therapeutic efficacy [116,135]. Approaches such as core cross-linking, polymer-drug conjugation, and stimuli-responsive cross-linkers have been developed to improve stability while preserving controlled drug release [116]. However, increasing structural sophistication may also complicate large-scale manufacturing, quality control, and regulatory evaluation. Future polymeric nanocarriers should therefore prioritize simple, reproducible designs that integrate biocompatibility, controlled degradation, structural stability, and scalable manufacturing without unnecessary complexity [144,145,146].

## 9. Conclusions and Future Perspectives

TNBC remains one of the most challenging breast cancer subtypes because of its molecular heterogeneity, aggressive progression, and lack of broadly applicable therapeutic targets. The unique biochemical characteristics of the TME provide multiple opportunities for responsive drug delivery, and advances in polymer chemistry have enabled the development of nanocarriers capable of exploiting these signals to improve tumor selectivity and therapeutic efficacy while reducing systemic toxicity.

Despite encouraging preclinical results, several barriers continue to limit clinical translation. One of the most important challenges is the spatial and temporal heterogeneity of the TME, which may result in inconsistent activation of stimulus-responsive systems within and between tumors. Furthermore, many nanocarriers are evaluated under simplified in vitro conditions or subcutaneous xenograft models that do not adequately reproduce the complexity of human TNBC. As a result, therapeutic performance observed in preclinical studies may not accurately predict clinical outcomes. Additional challenges include polymer stability, degradation behavior, long-term safety, scalable manufacturing, and regulatory approval.

Future research should focus on designing polymeric systems that respond to quantitatively defined TME characteristics observed in patients rather than idealized experimental conditions. Incorporating multiple stimuli into a single platform may further improve therapeutic precision, provided that each responsive element has a clear biological rationale and predictable activation profile. At the same time, integrating imaging or tracking functions could facilitate real-time evaluation of biodistribution, carrier activation, and treatment response, supporting both mechanistic studies and clinical translation.

Ultimately, successful translation will depend not only on advances in polymer engineering but also on a better understanding of TNBC biology and the variability of the tumor microenvironment. Bridging these two aspects will be essential for developing clinically relevant TME-responsive polymeric nanocarriers with reproducible performance and meaningful therapeutic benefit.

## Figures and Tables

**Table 1:** Comparative overview of TME-responsive polymeric nanocarriers for TNBC therapy: Stimuli, TME trigger, polymer/conjugate, responsive mechanism, key advantages, major limitations.

Stimulus	Trigger	Polymer/Conjugate	Responsive Mechanism	Key Advantages	Major Limitations	Reference
pH	Acidic extracellular TME and endosomal pH	HA; PEG-b-PDMAEMA-b-PDPA; PEI–PLA/PEG–PAsp; PEG-PLGA	Protonation of ionizable groups; cleavage of acid-labile bonds; polymer destabilization; acid-triggered PEG shedding	Tumor-selective drug release; enhanced cellular uptake; efficient endosomal escape; charge modulation	Heterogeneous tumor acidity; variable in vivo activation; potential toxicity associated with cationic components.	[55,56,57,58,59]
Redox	Elevated intracellular GSH	HA-TPGS; POEG-co-PVDSAHA; RGD-PEG-SS-DSPE; PBAE; DPA-DSDMA-PEG	GSH-mediated cleavage of disulfide/diselenide linkages; polymer degradation, micelle disassembly, or size reduction	Intracellularly selective activation; controlled payload release; enhanced tumor penetration; integration with targeting and resistance-modulating strategies	Heterogeneous intracellular GSH levels; excessively high GSH concentrations in some in vitro studies; limited long-term stability and biodegradation data	[70,71,72,73,74]
Hypoxia	Reduced oxygen level in poorly vascularized tumor regions	PLA-diazobenzene-PEG; mPEG–PDLA/PLLA–AZO; AZO-linked mPEG–PLL; Diazobenzene-linked mPEG–PLA	Bioreductive cleavage of azobenzene/diazobenzene or nitroaromatic linkers under hypoxia; carrier destabilization.	Selective activation in hypoxic niches; reduced off-target exposure; potential suppression of hypoxia-driven resistance.	Spatial heterogeneity of hypoxia; mismatch between trigger sensitivity and physiological oxygen levels	[88,89,90,91,92]
ROS	Elevated H_2_O_2_ and other reactive oxygen species	PLGA/PEI–β-cyclodextrin–ferrocene; PEG-b-PLA-azide/DTK; Peroxalate ester- polymer; Phenylboronic acid–containing polyacrylate	ROS-mediated cleavage of thioketal, peroxalate, or boronic ester linkages; polymer degradation.	Oxidative-stress-selective release; enhanced intracellular delivery; ROS amplification.	Spatial and temporal ROS heterogeneity; possible activation in inflammatory tissues.	[101,102,103,104,105]
Photo	Externally applied light stimulus; TME-associated hypoxia.	PS-PEG-COOH/PSMA; P(HEMA-Phe-NH_2_); MHBC/PNIPAM-AZO; HA-ANI	Photoisomerization, photochemical transformation, or photothermal conversion.	High spatiotemporal precision; on-demand activation; reduced off-target exposure; photodynamic/photothermal therapy.	Limited tissue penetration; dependence on external irradiation;	[112,113,114,115]
Temperature	Mildly elevated tumor temperature or externally applied hyperthermia	PNIPAM-co-PEGMA-co-AA;PNIPAAm-b-PNVCL; PLGA-PEG-PLGA; (P(MEO2MA-co-OEGMA-co-DMAEMA)-b-PLGA)); PNIPAM	LCST-mediated phase transition; changes in polymer hydration, conformation, nanoparticle state, or gelation.	Thermally triggered release; enhanced cellular uptake; sustained delivery; compatibility with photothermal therapy; improved therapeutic control.	Small tumor–normal temperature differential; dependence on external heating; heterogeneous thermal distribution; limited penetration of thermal stimuli.	[126,127,128,129,130]

## Data Availability

No new data were created or analyzed in this study. Data sharing is not applicable to this article.
